# Extracellular vesicles-derived miR-21 as a biomarker for early diagnosis and tumor activity in breast cancer subtypes

**DOI:** 10.1186/s40364-025-00724-y

**Published:** 2025-01-23

**Authors:** Claudia Omarini, Virginia Catani, Ilenia Mastrolia, Angela Toss, Federico Banchelli, Chrystel Isca, Daniele Medici, Ornella Ponzoni, Marco Brucale, Francesco Valle, Maria Cristina Baschieri, Roberto D’Amico, Valentina Masciale, Chiara Chiavelli, Federica Caggia, Carlo Augusto Bortolotti, Federico Piacentini, Massimo Dominici

**Affiliations:** 1 Division of Medical Oncology, Department of Oncology and Hematology, University Hospital of Modena, Modena, 41124 Italy; 2 Laboratory of Cellular Therapy, Department of Medical and Surgical Sciences for Children and Adults, University Hospital of Modena, Modena, 41124 Italy; 3 Division of Medical Oncology, Department of Medical and Surgical Sciences for Children and Adults, University Hospital of Modena, Modena, 41124 Italy; 4 Unit of statistical and methodological support to clinical research, University Hospital of Modena, Modena, 41124 Italy; 5 Division of Medical Oncology, Sant’Antonio Abate Hospital of Trapani, Erice, 91016 Italy; 6 Consiglio Nazionale delle ricerche (CNR), Istituto per lo studio dei materiali nanostrutturati (ISMN), Bologna, 40129 Italy; 7 Department of training, research and innovation, University Hospital of Modena, Modena , Italy; 8 Department of Life Sciences, University of Modena and Reggio Emilia, Modena, 41126 Italy

## Abstract

Emerging evidence highlights the key role of microRNA (miR)-21 in cell-to-cell communication and tumorigenesis. However, limited knowledge exists on the levels and clinical meaning of miR-21 in extracellular vesicles (EVs) of patients with breast cancer (BC). We assessed EV-derived miR-21 levels in one hundred women: 30 with early BC (EBC), 30 with metastatic BC on treatment progression (MBC), 30 cancer survivors on follow-up (FU) and 10 healthy donors (HD) as age- and body mass index (BMI)-matched controls. EVs isolated from serum samples were characterized using nanoparticle tracking analysis, scanning electron microscopy and atomic force microscopy to detect their concentration, size, morphology and mechanical properties. The levels of miR-21 in EVs was evaluated using real time PCR and compared between groups (EBC, MBC and FU vs. HD) by calculating the fold change and ΔΔCt statistic. EVs size and concentration did not differ significantly among patient groups. In the EBC group, the clinical stage at diagnosis and tumor subtype did not influence miR-21 levels. The levels of miR-21 were higher in the MBC group than in the HD group (*p* = 0.029), mainly in those who were human epidermal growth factor receptor 2 (HER2)+ (*p* = 0.0005) and hormone receptor-positive (*p* = 0.036). In particular, in the HER2 + subgroup, the miR-21 levels were significantly higher in those with active BC (both EBC and MBC) than in HDs (*p* = 0.002). Our findings suggest that miR-21 may be a promising biomarker for diagnosis and tumor activity, mainly in HER2 + BC.

**Supplementary Information:**

The online version contains supplementary material available at 10.1186/s40364-025-00724-y.

**To the editor**.

Detecting breast cancer (BC) and treatment resistance early remain significant unmet clinical needs [1]. Novel minimally invasive biomarkers could be extremely helpful in overcoming these issues and improving BC survival rates. Emerging evidence highlights the key role of microRNA (miR)-21 in cell-to-cell communication and tumorigenesis [2–5]. Consequently, miR-21 has recently been classified as an oncomiR [6]. Currently, contrasting and limited knowledge exists on the levels and clinical meaning of miR-21 isolated from extracellular vesicles (EVs) obtained from serum samples from patients with BC [7].

We conducted a clinical trial (ExoBreast), investigating the role of EVs circulating in the serum of BC patients, focusing on EV-derived miR-21 levels (Fig. 1A). We enrolled 100 women: 30 with early BC (EBC), 30 with metastatic BC on treatment progression (MBC), 30 cancer survivors on follow-up (FU), and 10 healthy donors (HDs) as age and body mass index (BMI)-matched controls (Fig. 1B). The clinical and pathological characteristics of the participant at the time of liquid biopsy are summarized in Table S1.

**Fig. 1:**
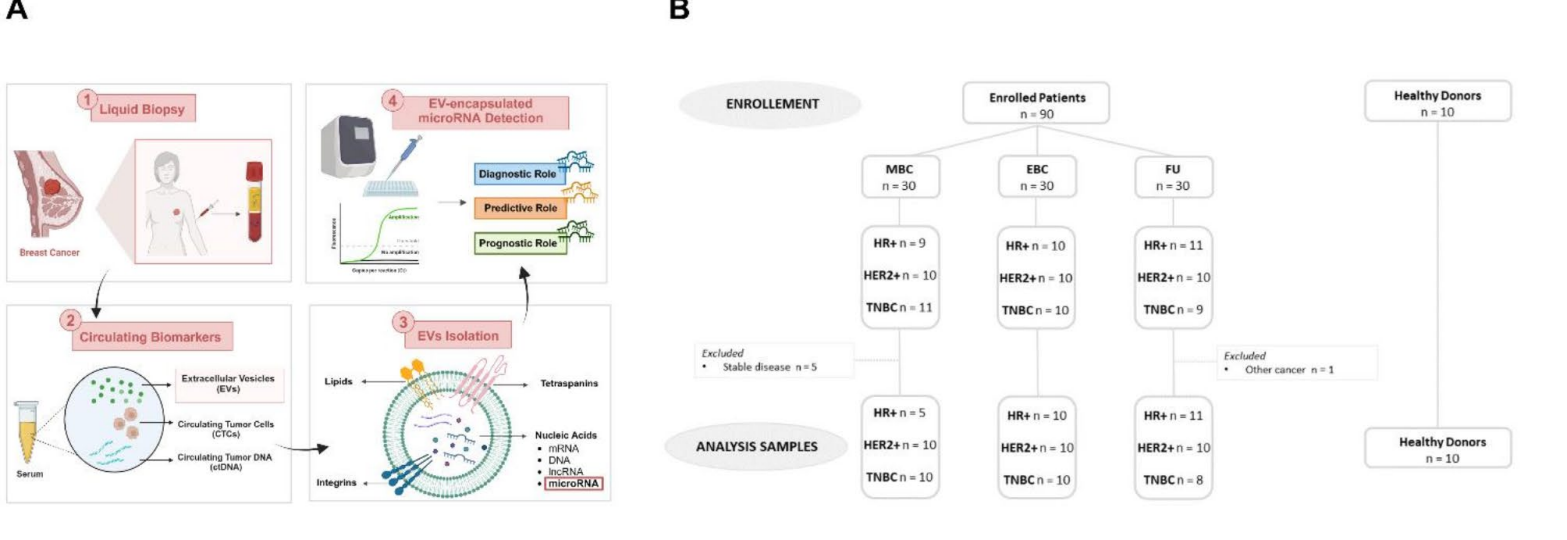
**(A)** A schematic representation of the project’s study design. **(B)** Flow chart of patient enrollment

Our team discovered that miR-21 levels were significantly higher in patients with MBC than in HDs (*p* = 0.029), mainly in those that were human epidermal growth factor receptor 2 (HER2)+ (*p* = 0.0005) and hormone receptor (HR)-positive (*p* = 0.036; Fig. 2). Firstly, after isolation from participants’ serum, all groups showed medium-small EVs with similar concentrations, sizes and shapes (Figures S1 and S2). In contrast, miR-21 levels differed among groups. In particular, miR-21 levels were higher in all BC groups than in the HD group at baseline: EBC (fold change [FC] = 1.266, 95% confidence interval [CI] = 2.082–0.770), MBC (FC = 1.758, 95% CI = 2.926–1.057), active BC (EBC and MBC; FC = 1.470, 95% CI = 2.360–0.916) and FU (FC = 1.292, 95% CI = 2.129–0.785; Fig. 2A, *y* = 1). However, the difference was only significant for the MBC group (*p* = 0.0299; Fig. 2A). Considering all patients with BC, there was no association between age (*p* = 0.293), menopausal state (*p* = 0.895), BMI (*p* = 0.426) and miR-21 levels. Considering the tumor subtype, in the HER2 + subgroup, miR-21 levels were significantly higher in patients with active BC (EBC and MBC) than in the HDs (*p* = 0.002; Fig. 2B). Regarding the HR + subgroup, miR-21 levels were only significantly higher in patients with MBC than in HDs (FC = 1.857, 95% CI = 3.320–1.039; *p* = 0.0367; Fig. 2C). No differences were detected in patients with triple-negative BC (TNBC; *p* > 0.05; Fig. 2D).

**Fig. 2:**
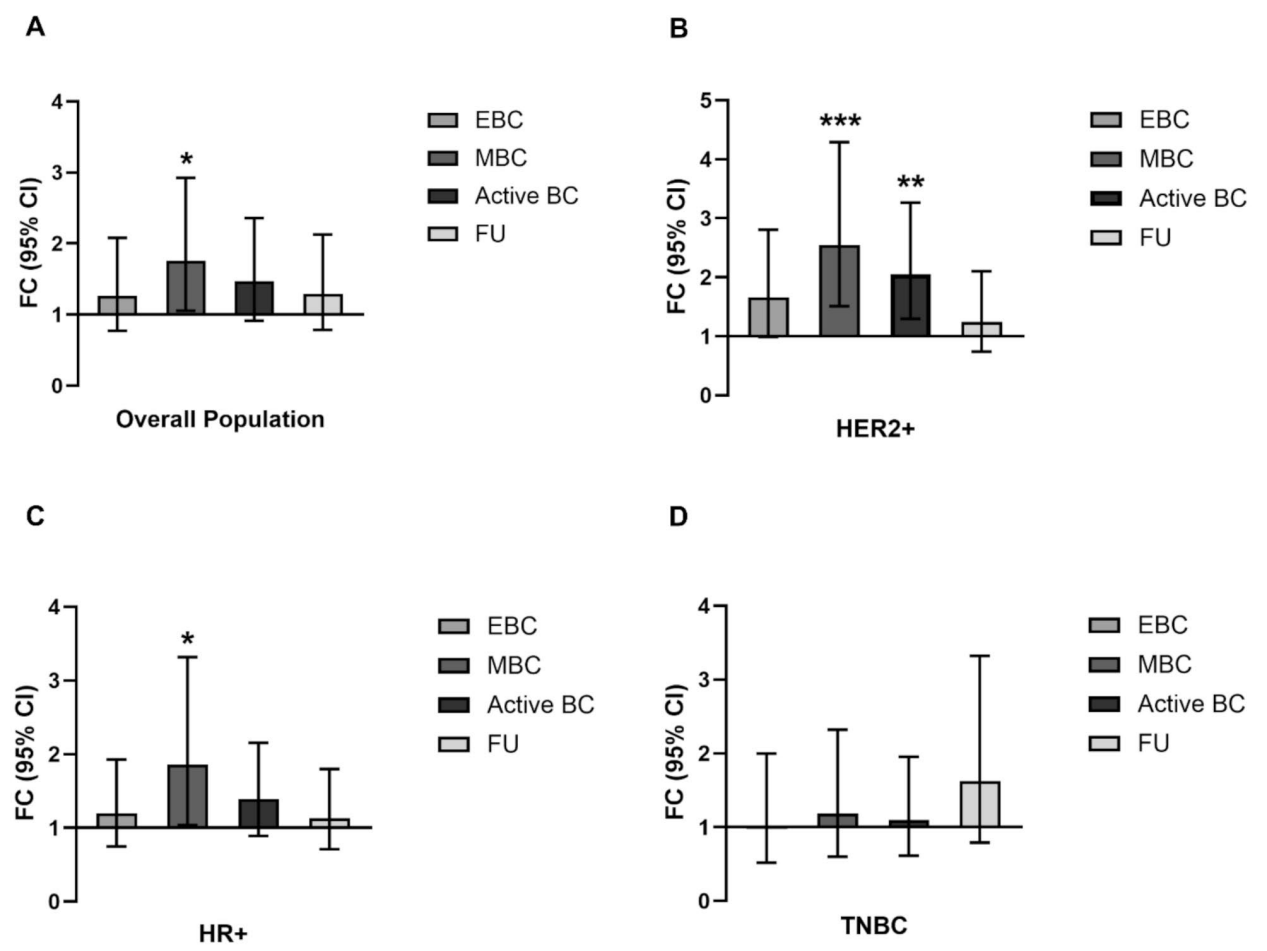
**(A)** miR-21 levels in all patients with BC, showing significantly higher levels in those with MBC than in HDs (*p* = 0.0299; *n* = 10 for HD; *n* = 30 for EBC; *n* = 25 for MBC; *n* = 29 for FU). **(B)** miR-21 levels in the HER2 + subgroup compared to HD group, showing significant differences for MBC (*p* = 0.0005) and active BC (*p* = 0.0022; *n* = 10 for HD, EBC, MBC and FU). **(C)** mir-21 levels in the HR + subgroup compared to the HD group, showing a significant difference for MBC (*p* = 0.0367; *n* = 10 for HD, *n* = 10 for EBC, *n* = 5 for MBC, and *n* = 11 for FU). **(D)** mir-21 levels in the TNBC subgroup compared to the HD group, showing no significant difference (*p* > 0.05; *n* = 10 for HD, *n* = 10 for EBC, *n* = 10 for MBC, and *n* = 8 for FU)

Our data suggest that miR-21 may be a promising biomarker for diagnosis and tumor activity, mainly in patients with HER2 + BC. The ExoBreast trial found higher levels of EV-derived miR-21 in patients with HER2 + or HR+/HER2- MBC than in HDs. Regarding MBC, only patients with progressing disease were included in the analysis, since circulating miR-21 was previously associated with BC proliferation and progression [8–11]. Our findings confirm earlier data on higher exosomal miR-21 levels in patients with BC than HDs [12, 13]. Interestingly, miR-21 levels were significantly higher in HER2 + tumors than in HDs, even when jointly considering EBC and MBC. Therefore, considering only the subgroup, our study revealed significantly different miR-21 levels between patients with BC and HDs.

Since evidence on the association between miR-21 levels and BC molecular subtype is still conflicting, pre-clinical in vitro models have been considered. To our knowledge, our study is one of the largest evaluating EV-derived miR-21 levels as a biomarker for BC detection and one of the few reporting the clinical-pathologic characteristics of enrolled patients with BC. Moreover, since EV levels may vary according to age and BMI, our case population was matched for age and BMI with HDs [14, 15]. Our data suggest that miR-21 may be a promising biomarker for diagnosis and tumor activity, mainly in patients with HER2 + BC. In patients with HER2 + BC, miR-21 has shown utility in cancer detection (active BC subgroup), and in monitoring evolution to follow patients in real time. Further information from basic and clinical research is required before EV-derived miR-21 may be introduced as a biomarker.

## Electronic supplementary material

Below is the link to the electronic supplementary material.

Supplementary Material 1

## Data Availability

No datasets were generated or analysed during the current study.

## Abbreviations

miR-21: microRNA-21
EVs: Extracellular vesicles
BC: Breast cancer
EBC: Early breast cancer
MBC: Metastatic breast cancer
FU: Follow up
HD: Healthy donor
BMI: Body max index
HR+: Hormone receptor positive
HER2+: Human epidermal growth factor receptor positive
TNBC: Triple negative breast cancer
CI: Confidence interval.
