# Supplementary material for: Extracellular vesicles-derived miR-21 as a biomarker for early diagnosis and tumor activity in breast cancer subtypes
Source: Biomark Res. 2025 Jan 23;13:14. doi: 10.1186/s40364-025-00724-y (PMC11756162; doi:10.1186/s40364-025-00724-y)
Supplement: Supplementary file 1 — Supplementary Material 1 [file 40364_2025_724_MOESM1_ESM.docx]

**Additional files**

**Materials and Methods**

**Patient population**

The ExoBreast trial included 100 enrolled women: 90 patients with breast cancer (BC) and 10 healthy donors (HDs). The patients were divided into three groups according to the BC setting: 30 had early BC (EBC), 30 had metastatic BC (MBC), and 30 were cancer survivors on follow-up (FU; Figure 2). The enrolled HDs were for age-, menopausal status- and body mass index (BMI)-matched with the patients with BC. Tumor subtypes were defined as follows: human epidermal growth factor receptor 2 (HER2)+ BC (HER2 status was reported as positive or negative according to immunochemistry [IHC] 3+ or in situ hybridization [ISH] amplification), hormone receptor (HR)+ BC (estrogen and/or progesterone receptors expression >1% and HER2-) and triple-negative BC (TNBC; HR- and HER2-). In particular, the EBC group included patients with stage I–III BC: 10 HR+, 10 HER2+ and 10 TNBC. The MBC group included patients with stage IV disease on progression at enrollment: 9 HR+, 10 HER2+ and 11 TNBC. Finally, FU group included patients with previous EBC diagnosis who had ended systemic adjuvant treatment (only adjuvant endocrine therapy was allowed). Notably, five patients in the MBC group were excluded from the analysis due to stable disease at the computed tomography (CT) scan performed after enrollment, while one patient in the FU cohort was excluded due to a subsequent gastric cancer diagnosis. Participants’ clinical and tumor characteristics at enrollment are listed in Table 1. Regarding the tumor stage in the EBC cohort, we considered the pathological stage for patients who underwent primary surgery and the clinical stage for patients who received neo-adjuvant chemotherapy.

**Serum collection and EV isolation**

Peripheral blood samples were collected from patients with BC and HDs in 10 cc clot activator, silicone-coated tubes (Vacutainer serum tubes; Becton Dickinson, Franklin Lakes, NJ, USA) and allowed to clot for 30 minutes at room temperature (RT). The serum was obtained by centrifugation at 2,000 x *g* and RT for 10 minutes, and then aliquoted and stored at -80°C until needed.

Extracellular vesicles (EVs) were isolated using the commercial kit Total Exosome Isolation Kit from serum (Invitrogen, Waltham, MA, USA), according to the manufacturer’s instructions. Briefly, each 500 µL sample aliquot was initially centrifuged at 2,000 x *g* and RT for 30 minutes to remove cells and debris. Next, the corresponding amount of reagent was added proportionally to the starting sample volume, according to the manufacturer’s instructions. Then, the mixture was vortexed and incubated at 4°C for 30 minutes, then centrifuged at 10,000 x *g* and RT for 10 minutes to precipitate EVs. Finally, the EV pellet was resuspended in 100 µL of nuclease-free water for NTA characterization and then stored at -80°C for subsequent analyses.

**Nanoparticle tracking analysis (NTA)**

NTA was performed using the ZetaView PMX 110 (Particle Metrix, Meerbusch, Germany) and its corresponding software (ZetaView 8.05.11 SP4). Briefly, 1 mL of the sample, diluted in nuclease-free water, was loaded into the cell. For each measurement, two cycles were performed by scanning 11 cell positions each and capturing 30 frames per second under the following settings: Focus = autofocus, Camera sensitivity for all samples = 80.0, Shutter = 100, Scattering Intensity = detected automatically, Cell temperature = 25°C. After capture, EV size and concentration were determined using the built –in ZetaView Software with the following analysis parameters: maximum particle size = 1000, minimum particle size = 5, minimum particle brightness = 25. Polystyrene particles from Particle Metrix with a known average size of 100 nm were used to calibrate the instrument before sample readings.

**Scanning Electron Microscopy (SEM)**

SEM was employed to visually confirm the presence of EVs in the resuspended pellet. The EV samples were prepared for SEM analysis by first centrifuging the EVs isolated from serum at 800 x *g* and RT for 2 minutes, then centrifuging the supernatant at 20,000 x *g* and 4°C for 40 minutes. Next, the pellet was fixed with 4% paraformaldehyde at 4°C for 1 hour and washed twice with nuclease-free water. The morphology of the EVs was examined using FEI Nova NanoSEM™ 450 scanning electron microscopes (ThermoFisher Scientific, Waltham, MA, USA). Before SEM imaging, the samples were coated with a 10 nm layer of gold by applying a current of 25 mA for 1 minute. Imaging was conducted with the TLD detector mode at magnifications from 25,000× to 200,000×.

**Atomic Force Microscopy (AFM)**

AFM micrographs were recorded following a procedure described elsewhere (1). Images were acquired in PeakForce mode on a Multimode 8 microscope (Bruker, Billerica, MA, USA) equipped with Scanasyst Fluid+ probes (Bruker), a Nanoscope V controller, a type JV piezoelectric scanner and a sealed fluid cell. Briefly, EVs were deposited on poly-L-lysine functionalized glass coverslips and left to adsorb for 30 minutes at 4°C, then inserted in the fluid cell without further rinsing. Sample dilution was adjusted in successive depositions to maximize the number of isolated objects. Quantitative morphometry was performed via Gwyddion 2.61 and custom Python scripts. Two parameters were calculated for each object deposited on the surface: its original diameter in solution and the contact angle (CA) it displayed once adsorbed on the surface. The latter was previously demonstrated to be directly proportional to the mechanical stiffness of intact EVs (1,2).

**Extraction of EV-derived miRNAs**

EV-derived miRNAs were extracted using the Total Exosome RNA and Protein Isolation Kit (Invitrogen) according to the manufacturer’s instructions. Briefly, the organic extraction phase was followed by an enrichment phase to obtain the small RNAs retained by the filter, washed through passages with two wash solutions containing ethanol and recovered in the last elution step with nuclease-free water. Next, 2 µL of the miRNA sample was reverse-transcribed into cDNA using the TaqMan Advanced miRNA cDNA Synthesis kit (Invitrogen) according to the manufacturer’s instructions. Briefly, an initial poly(A) tailing reaction was conducted at 37°C for 45 minutes and 65°C for 10 minutes. Next, an adaptor ligation reaction was conducted at 16°C for 60 minutes. Then, a reverse transcription reaction was conducted at 42°C for 15 minutes, followed by 85°C for 5 minutes. Next, a miRNA amplification reaction was conducted as follows: 95°C for 5 minutes, followed by 14 cycles of denaturation at 95°C for 5 minutes and annealing and extension at 95°C for 3 seconds. The final step consisted of a reaction at 99°C for 10 minutes to obtained the final product.

**MiR-21 quantification by real-time PCR**

Quantitative real-time PCR was performed using the QantStudio^TM^1 Real-Time PCR System (Applied Biosystems, Waltham, MA, USA) and the TaqMan Fast Advanced Master Mix (Invitrogen). Each PCR reaction consisted of 2.5 µL of 1:10 diluted cDNA template in a 10 uL total volume. The miR-21 concentration was quantified using TaqMan Advanced miRNA Assays (Invitrogen) and miR-16 as the endogenous control. The amplification program began at 95°C for 20 seconds, followed by 40 cycles of denaturation at 95°C for 1 second and annealing and extending at 60°C for 20 seconds.

**Statistical analysis**

Categorical variables were described as the absolute and percentage frequencies, and numerical variables were described as the median and range. The characteristics were compared between each BC group and HD group using the Wilcoxon rank-sum test for numerical variables and Fisher’s exact test for categorical variables. The expression of miR-21 was compared between groups by calculating the ΔΔCt statistic and the fold change (FC, equal to 2^-ΔΔCt^), considering miR-16 as the housekeeping gene. The ΔΔCt statistic was calculated using a linear mixed model approach, considering cycle thresholds as the dependent variable, and gene type (miR-21 or miR-16), group (EBC, MBC and FU vs. HD), and their interaction term as the independent variables (3). The model was also adjusted for age, BMI, and menopausal status to account for their potential confounding effects. Random intercept and random slope terms were included in the mixed model to account for individual variability in average miR-21 and miR-16 cycle thresholds. Uncertainty in estimates was reported as the 95% confidence interval (CI). Analyses were performed using the R statistical software (version 4.3.2; The R Foundation for Statistical Computing, Wien, Austria). A *p* < 0.05 was considered statistically significant.

**Supplementary Figure**

**Figure S1. The** nanoparticle tracking analysis (NTA) and morphological characterization of EVs. (**A**) Representative NTA profile of the HDs and patients with BC and (**B**) the EV concentration (particles/mL) and size distribution (nm) expressed as the mean ± standard deviation, showing no significant differences among groups. (**C**) Representative morphology of EVs by scanning electron microscopy (SEM) analysis.


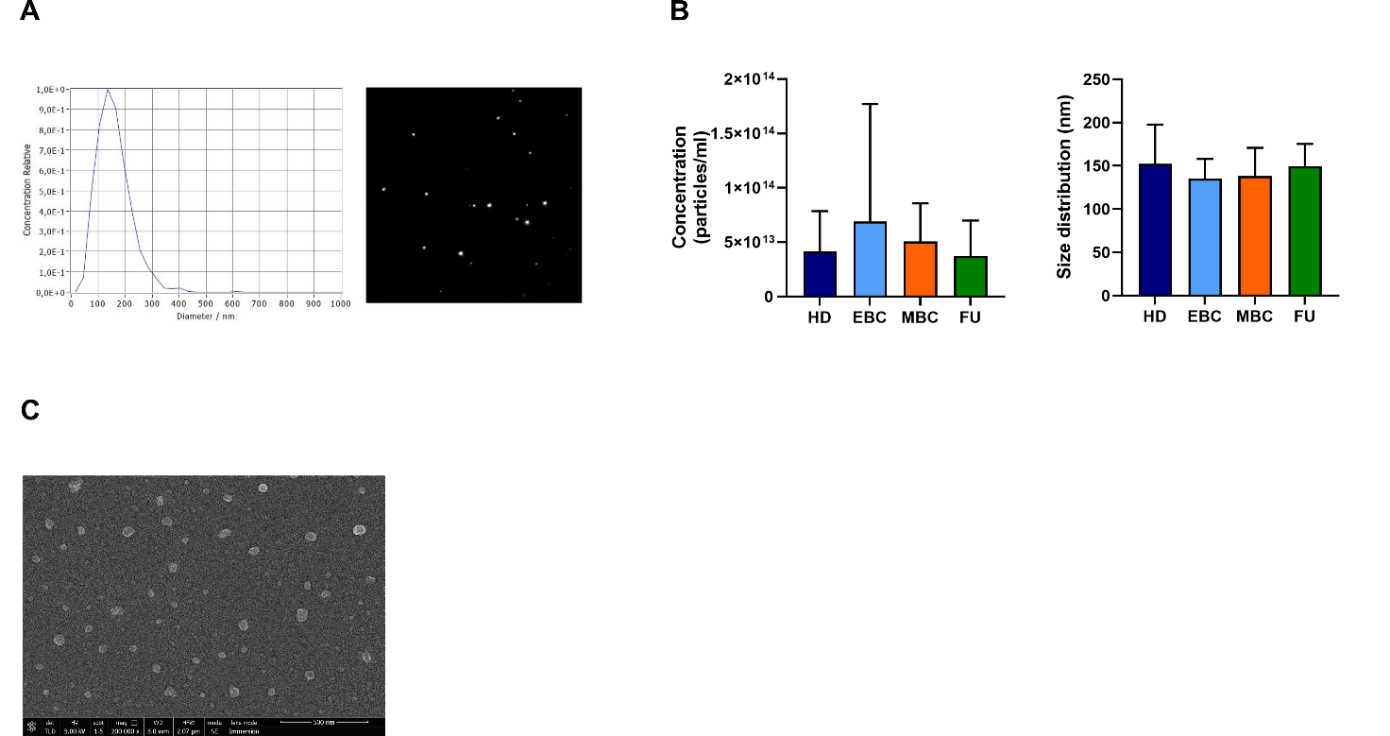


**Figure S2.** Atomic force microscopy (AFM) analysis. (A) A representative image of AFM visualization in 2D and 3D rendering (5 × 5 µm; scale = bar 1µm). (B) A representative scatter plot of contact angle (CA) vs. diameter shows overlapping clusters between groups, without significant differences. Limiting the analysis to putatively intact EVs with diameters >50 nm, (C) size and (D) CA distributions did not differ significantly among groups.

*
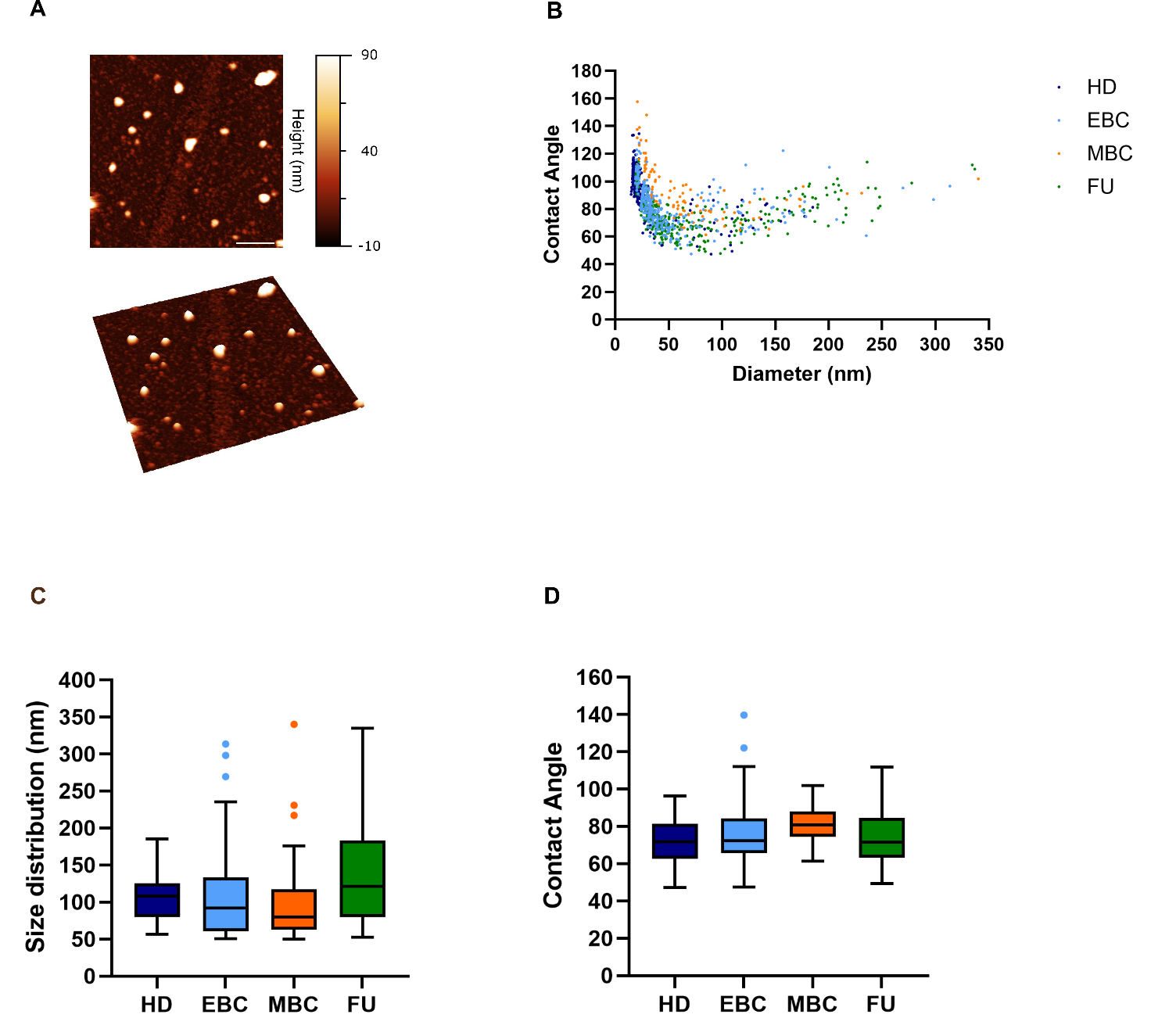
*

**Supplementary Table**

**Table S1.** Participants’ clinical and pathological characteristics at the time of liquid biopsy.

|  | MBC  n=25 | *p* | EBC  n=30 | *p* | FU  n=29 | *p* | HD  n=10 |
| --- | --- | --- | --- | --- | --- | --- | --- |
| **Median age**  **(range)** | 55 yrs  (33-74) | 0.351 | 53 yrs  (35-76) | 0.542 | 51 yrs (37-72) | 0.847 | 49 yrs (36-65) |
| **Menopausal state** |  |  |  |  |  |  |  |
| Pre- | 10 | 0.712 | 18 | 0.717 | 15 | 1.000 | 5 |
| Post- | 15 |  | 12 |  | 14 |  | 5 |
| **BMI (body mass index)** |  |  |  |  |  |  |  |
| <26 | 17 | 0.738 | 16 | 0.543 | 20 | 0.731 | 7 |
| ≥ 26-30 | 3 |  | 6 |  | 3 |  | 2 |
| ≥ 30 | 5 |  | 8 |  | 6 |  | 1 |
| **Co-morbidities** |  |  |  |  |  |  |  |
| Cardiovascular diseases | 9 | 0.218 | 7 | 0.653 | 2 | 1.000 | 1 |
| Metabolic disorders | 6 | 0.152 | 5 | 0.306 | 2 | 1.000 | 0 |
| Previous cancer | 2 | 1.000 | 3 | 0.560 | 0 | 1.000 | 0 |
| Rheumatological disease | 0 | 1.000 | 1 | 1.000 | 0 | 1.000 | 0 |
| **Breast Cancer Biology** |  |  |  |  |  |  |  |
| HR + | 5 | *NA* | 10 | *NA* | 11 | *NA* | |
| HER2+ | 10 |  | 10 |  | 10 |  |  |
| TNBC | 10 |  | 10 |  | 8 |  |  |
| **Treatment's line** |  |  |  |  |  |  |  |
| Before any treatment | 3 | *NA* | 30 | *NA* | | | |
| 1st | 5 |  | 0 |  |  |  |  |
| 2nd | 5 |  | 0 |  |  |  |  |
| 3rd or more | 12 |  | 0 |  |  |  |  |
| **Clinical/Pathological stage** |  |  |  |  |  |  |  |
| I  (pT1pN0/mic M0) | 0 | *NA* | 9 | *NA* | | | |
| II  (pT1pN1 or pT2pN0-1 or pT3pN0 M0) | 0 |  | 13 |  |  |  |  |
| III  (pT3pN1 or pT1-4pN2-3 M0) | 0 |  | 8 |  |  |  |  |
| IV  (any pT any pN M1) | 25 |  | 0 |  |  |  |  |
| **Metastatic sites** |  |  |  |  |  |  |  |
| Visceral | 19 | *NA* | | | | | |
| Non visceral | 2 |  |  |  |  |  |  |
| Brain | 4 |  |  |  |  |  |  |

*p* = p-value vs. HDs

*NA* = not applicable

**References**

1. Ridolfi A, Brucale M, Montis C, Caselli L, Paolini L, Borup A, et al. AFM-Based High-Throughput Nanomechanical Screening of Single Extracellular Vesicles. Anal Chem. 2020 Aug 4;92(15):10274–82.

2. Ridolfi A, Conti L, Brucale M, Frigerio R, Cardellini J, Musicò A, et al. Particle profiling of EV-lipoprotein mixtures by AFM nanomechanical imaging. J Extracell Vesicles. 2023 Oct;12(10):e12349.

3. Steibel JP, Poletto R, Coussens PM, Rosa GJM. A powerful and flexible linear mixed model framework for the analysis of relative quantification RT-PCR data. Genomics. 2009 Aug;94(2):146–52.
